# Faculty and student perceptions of the feasibility of individual student–faculty meetings

**DOI:** 10.1007/s40037-012-0011-6

**Published:** 2012-04-04

**Authors:** B. F. Mulder, M. H. Erich, J. C. C. Borleffs, A. F. Elgersma, J. Cohen-Schotanus

**Affiliations:** Center for Research and Innovation in Medical Education, University of Groningen and University Medical Center Groningen, P. O. Box 196, 9700 AD Groningen, the Netherlands

**Keywords:** Undergraduate medical education, Faculty, Students, Social and academic involvement

## Abstract

The extent to which students feel involved in their education positively influences academic achievement. Individual student–faculty meetings can foster student involvement. To be effective, faculty acknowledgement of the benefit of these meetings is a prerequisite. The aim of this study was to explore faculty perceptions of individual student–faculty meetings. In addition we investigated students’ perceptions. As part of the undergraduate programme, mandatory individual intake and follow-up meetings between first-year medical students (*n* = 425) and senior faculty members (*n* = 34) have been implemented from 2009 onwards. We administered a questionnaire on faculty perceptions of the benefit and impact of intake meetings. Subsequently, after both meetings had been held, strong and weak points of the mandatory programme were explored using open-ended questions. Students’ perceptions were investigated by open-ended questions as a part of the curriculum evaluation process. Faculty enjoyed the meetings (90 %), perceived the meetings to be beneficial (74 %) and expected a positive effect on student involvement (74 %). Faculty appreciated the opportunity to give advice tailored to students’ personal needs and levels of performance. The students appreciated the meetings and the attention given to their personal situation and study progress. Faculty and student appreciation of the meetings seems to support the assumption that the individual meetings increase students’ social and academic involvement. Further research should focus on the impact of individual student–faculty meetings on students’ learning behaviours.

## Introduction

Academic achievement is influenced by the extent to which students feel involved in their own education. According to Tinto’s theoretical model of integration, students are more likely to persist in college when they feel part of it [1]. The more students act and feel socially and academically involved, the less likely they are to drop out and the higher their study success, and levels of learning and development. Regular student–faculty interactions are conducive to student involvement [2–4].

Nowadays, problem-based learning (PBL) curricula are well established in health education [5]. Within PBL curricula, small-group activities provide several opportunities for student–faculty interaction; however, these meetings mainly concentrate on knowledge acquisition and less on individual well-being. To enhance student involvement, individual student–faculty meetings might be a useful addition. In accordance with Tinto’s theory, such meetings increase the frequency of one-on-one student–faculty interaction and might affect students’ commitment to and responsibility for their own learning, both prerequisites for PBL.

For such meetings to be effective, faculty should acknowledge their usefulness. Otherwise, faculty might show resistance to conducting the meetings which, in turn, might lead to lower performance [6], as is explained by job satisfaction theories [7]. Therefore, we focused our study on faculty perceptions of individual student–faculty meetings. To establish a broad image of the individual meetings, we also explored students’ perceptions.

## Method

### Context

The undergraduate medical curriculum of the University of Groningen comprises a three-year preclinical Bachelor’s programme and a 3-year clinical Master’s programme. Based on the PBL concept, small-group sessions are scheduled throughout the curriculum.

### Procedure

As of 2009, mandatory individual intake and follow-up meetings between first-year students and faculty are a part of our first-year curriculum. The meetings were designed to be in line with Tinto’s theory as well as our curriculum. Faculty who met specific prerequisites were asked to participate in the programme. The prerequisites were: seniority, academically qualified, a central position in our curriculum or educational organisation and not appointed as a student counsellor. All faculty who were asked to participate did so. Faculty were trained in the use of both meeting protocols during 2-hour training sessions for each protocol.

The students were randomly assigned to faculty. The intake meetings were held during the first 2 weeks of the first semester and follow-up meetings—between the same faculty member and student at the start of the second semester. Both meetings lasted 30 min with 15 extra minutes allocated for preparation and administration.

During the intake meetings, students and faculty discussed the confidentiality agreement and the institutional code of conduct, and the students signed their consent. They also discussed their mutual expectations and assignments to complete a questionnaire about themselves and their history and to make a study plan for the first semester—the students had prepared in advance. Afterwards, the students had to draw up the minutes of the meeting and send these to faculty for approval.

During the follow-up meetings, students and faculty discussed the students’ progress during the first semester and their study plans for the second semester. Depending on student progress, the emphasis of the meetings was either on improving study behaviour or on possibilities for extracurricular activities.

### Participants

All 34 faculty members who participated in the programme were invited to complete the questionnaire. Of these faculty members, 30 % had obtained a Master’s degree, 41 % had obtained a PhD and the remaining 29 % were professors. Of participating faculty, 35 % were medical doctors. The other faculty members had backgrounds in life and natural sciences (30 %) or behavioural and social sciences (35 %). In total, faculty time investment in the programme was 773.5 h (34 × 4 training hours and 1.5 × 425 h for the meetings).

Students’ perceptions (*n* = 425) of the meetings were part of the curriculum evaluation process of the first study year.

### Instrument

After the intake meetings, participating faculty were asked to complete an anonymous questionnaire. This questionnaire contained ten statements about the benefit, set-up and expected impact of intake meetings and the students spoken to, using a 5-point Likert scale for responses (++ totally agree; − totally disagree) (Table 1). After both meetings, strong and weak points of the individual meeting programme were explored using open-ended questions.

**Table 1:** Responses to intake meeting questionnaire

	++/+	±	−/−−
Benefit of meetings			
Intake meetings with first-year students are useful	74 %	19 %	6 %
Interpretation of the set-up of the meeting			
The meeting protocol was a good tool for the meeting	77 %	16 %	6 %
I gave my own interpretation to the meetings	74 %	16 %	10 %
I really enjoyed the intake meetings	90 %	10 %	0 %
Perception on students spoken to			
The questionnaire and the assignment enabled me to form a good picture of the student	61 %	32 %	6 %
Students took the intake meetings seriously	87 %	13 %	0 %
Students are aware of the rules in their first-year	23 %	32 %	45 %
Expected impact of the meetings			
I think that I have influenced the study behaviours of the students I met	35 %	55 %	10 %
The intake meetings helped students feel part of the degree programme	74 %	23 %	3 %
The intake meetings will have a positive effect on the success rate of the propaedeutic phase.	26 %	61 %	13 %

Students’ perceptions were investigated by open-ended questions concerning the strong and weak points of the individual meeting programme.

## Findings

The questionnaire was completed by 31 faculty members (91 %). In general, they enjoyed the meetings (90 %), perceived the meetings to be beneficial (74 %) and expected a positive influence on student involvement (74 %). Faculty were more hesitant about the meetings having a positive effect on study behaviour (35 %) and study progress (26 %). Faculty found that students were not well aware of the examination rules (45 % −/−−) (Table 1).

Positive aspects emerging from the open-ended questions were: the 30 min’ duration of the meetings, obligatory attendance, the questionnaire students had to complete prior to the meeting, the signing of the agreements, the dual set-up (intake and follow-up) and the focus on both the students’ personal needs and their study plans. Topics for improvement were the number of students allocated to an individual faculty member and the strictness of the protocol.

Of the students, 70 % responded to the evaluation. Positive aspects mentioned by the students were: the personal attention given, advice tailored to their individual situation, the objective view on their study behaviour and progress, the idea that someone is keeping an eye on their progress, and the explicit opportunity to ask all kinds of questions, especially at the start of their studies. Topics for improvement were the agenda of the follow-up meeting with regard to well-performing students and attention for explicit training of study skills.

## Discussion

We investigated faculty perceptions of the individual student–faculty meetings, because faculty acknowledgement is a prerequisite for the success of such meetings. Faculty appreciated the individual meetings, perceived these meetings as beneficial and expected a positive influence of the meetings on student involvement. The main reason for perceiving the meetings as beneficial was that they offered an opportunity to refer to students’ unique personal situations and give advice tailored to the students’ personal needs and levels of performance. However, not all faculty members agreed with the statements on study behaviour and graduation rates, maybe because they were not able to predict whether the students would follow their advice. In this study we did not investigate how the students acted on the advice given. Further research is needed on the influence of faculty’s advice on students’ study behaviours.

In line with faculty perceptions, the students appreciated the personal attention and the advice tailored to their personal needs and levels of performance, especially during the intake meeting. Their responses indicate an increased level of involvement, because all the strong points of the programme they mentioned indicate appreciation for being noticed by the organisation.

Obviously, students may also consult a student counsellor without faculty intervention. However, most students are not aware of the help available to them, especially at the beginning of their study. Moreover, students most in need of assistance are often reluctant to seek help [8]. The mandatory character of the individual meetings obviates this potential avoidance.

A possible drawback of the programme might be the amount of time a faculty member needs to invest. Given faculty suggestions for change, we advise restricting the number of students allocated to a faculty member to ten.

We increased the student–faculty interaction in our curriculum by implementing mandatory individual intake and follow-up meetings between first-year students and faculty. According to Tinto’s theoretical model of integration regular student–faculty interactions are conducive to student involvement and student involvement is conducive to study success and levels of learning and development [1–4]. Our study revealed that faculty acknowledged the usefulness of our programme, which is a prerequisite for its success. Faculty and student appreciation of the meetings seems to support the assumption that the individual meetings increase students’ social and academic involvement.
